# The identification of novel immunogenic antigens as potential *Shigella* vaccine components

**DOI:** 10.1186/s13073-020-00824-4

**Published:** 2021-01-15

**Authors:** Ruklanthi de Alwis, Li Liang, Omid Taghavian, Emma Werner, Hao Chung The, Trang Nguyen Hoang Thu, Vu Thuy Duong, D. Huw Davies, Philip L. Felgner, Stephen Baker

**Affiliations:** 1 Program in Emerging Infectious Diseases, Duke-NUS Medical School, Singapore, Singapore; 2 Viral Research and Experimental Medicine Centre, SingHealth Duke-NUS Academic Medical Centre, Singapore, Singapore; 3 Vaccine Research & Development Center, School of Medicine, University of California Irvine, Irvine, CA USA; 4 Laboratory of Molecular Biology, Cambridge Biomedical Campus, Cambridge, UK; 5 The Hospital for Tropical Diseases, Wellcome Trust Major Overseas Programme, Oxford University Clinical Research Unit, Ho Chi Minh City, Vietnam; 6 Cambridge Institute of Therapeutic Immunology & Infectious Disease (CITIID), Level 5, Jeffery Cheah Biomedical Centre, Cambridge Biomedical Campus, University of Cambridge, Cambridge, CB2 0AW UK

## Abstract

**Background:**

*Shigella* is a major diarrheal pathogen for which there is presently no vaccine. Whole genome sequencing provides the ability to predict and derive novel antigens for use as vaccines. Here, we aimed to identify novel immunogenic *Shigella* antigens that could serve as *Shigella* vaccine candidates, either alone, or when conjugated to *Shigella* O-antigen.

**Methods:**

Using a reverse vaccinology approach, where genomic analysis informed the *Shigella* immunome via an antigen microarray, we aimed to identify novel immunogenic *Shigella* antigens. A core genome analysis of *Shigella* species, pathogenic and non-pathogenic *Escherichia coli*, led to the selection of 234 predicted immunogenic *Shigella* antigens. These antigens were expressed and probed with acute and convalescent serum from microbiologically confirmed *Shigella* infections.

**Results:**

Several *Shigella* antigens displayed IgG and IgA seroconversion, with no difference in sero-reactivity across by sex or age. IgG sero-reactivity to key *Shigella* antigens was observed at birth, indicating transplacental antibody transfer. Six antigens (FepA, EmrK, FhuA, MdtA, NlpB, and CjrA) were identified in in vivo testing as capable of producing binding IgG and complement-mediated bactericidal antibody.

**Conclusions:**

These findings provide six novel immunogenic *Shigella* proteins that could serve as candidate vaccine antigens, species-specific carrier proteins, or targeted adjuvants.

**Supplementary Information:**

The online version contains supplementary material available at 10.1186/s13073-020-00824-4.

## Background

*Shigella* is the causative agent of shigellosis, a severe acute gastrointestinal infection that frequently presents as bloody diarrhea, fever, and severe abdominal pain [1]. In 2016, *Shigella* was estimated to cause > 250 million cases and > 200,000 deaths globally [2]. Higher income countries experience *Shigella* infections among travelers, aging populations, deployed military personnel, and men who have sex with men (MSM) [2, 3]. However, the preponderance of the *Shigella* disease burden is in children aged under 5 years residing in low-middle income countries (LMICs). Infection in this vulnerable group can also result in significant long-term consequences such as severe stunting and impaired cognitive development [2, 4]. The global epidemiology of *Shigella* is worsened by the emergence and spread of multi- and extensively drug resistant (MDR and XDR) variants, making infections increasingly difficult to treat [5]. The principal method of *Shigella* prevention has been improvements in water, sanitation, and hygiene (WASH) [6]. However, due to the low infectious dose, the standard of WASH required to break transmission is difficult to attain in many LMICs [7]. Furthermore, recent application of molecular techniques to identify *Shigella* infections found a severe underestimation of the global *Shigella* burden [8, 9], highlighting the need for new low-cost prevention techniques.

There is currently no licensed vaccine against *Shigella* [10]. However, studies in animals and controlled human infection models (CHIMs) have shown that protection through immunization is feasible [11, 12]. Natural disease epidemiology in humans and non-human primate infection studies show complete protection from re-infection with a homologous *Shigella* species. Long-term homologous protection has been attributed to serotype-specific systemic (serum IgG) and mucosal (IgA) antibody responses [12, 13]. The most immunodominant target of the *Shigella* IgG and IgA response is the O-antigen component of lipopolysaccharide (LPS) [14, 15]. LPS/O-antigen-specific antibodies elicit protection through antibody-mediated opsonization, phagocytosis, and intracellular cytotoxicity [13]. However, antibodies against *Shigella* O-antigen are highly specific for the infecting species only [11, 13], and do not provide protection against heterologous *Shigella* species. Since the *Shigella* genus consists of four species and > 50 serotypes, a lack of cross-protection against heterologous species and serotypes poses a major challenge for vaccine development [16]. This challenge may be overcome by a vaccine that elicits either a broadly reactive immune response or numerous species-specific responses against the globally dominant *Shigella* species (i.e., *S. flexneri* and *S. sonnei*) [17].

The primary strategy of developing an efficacious *Shigella* vaccine has been to elicit antibody responses targeting *Shigella* O-antigen [10]. Live-attenuated vaccines with genetically attenuated *Shigella* [18–20], or the expression of *Shigella* O-antigen on live-attenuated vectors [21], have been shown to induce good antibody responses against O-antigen. Multivalent killed-vaccines also induce high titers of serum IgG and mucosal IgA targeting *Shigella* O-antigens and have shown protection in early clinical development [22, 23]. Recombinant forms of *Shigella* O-antigen have also been pursued as vaccine candidates [10], with a *Shigella* O-antigen conjugated to a carrier protein [24–26], which engages T cell help and produces a longer lasting antibody response to the polysaccharide antigen [27]. Various immunogenic proteins, such as the toxins from other pathogens, have been used as carrier proteins [28, 29]. However, protein antigens from *Shigella* have not been evaluated as a carrier protein for *Shigella* O-antigen.

Whole genome sequencing provided the ability to predict and derive novel antigens for use as vaccines, and this approach ultimately gave rise to the meningococcal B vaccine [30–32]. Further technological advances in immunology and protein engineering to study the interaction of pathogens with the immune system can aid in reverse engineering of protective immunogens [33–35]. Here, we aimed to identify novel immunogenic *Shigella* antigens that could serve as *Shigella* vaccine candidates, either alone, or when conjugated to *Shigella* O-antigen. Therefore, we conducted immunogen prediction using bioinformatic analysis, then created a protein microarray of predicted immunogenic *Shigella* antigens. These expressed antigens were screened for immunogenicity using polyclonal antibodies from patients who recovered from confirmed *Shigella* infections, to identify a novel set of proteins which may facilitate the development of novel *Shigella* vaccines.

## Methods

### Ethics

Human serum samples for the purposes of this investigation were collected from an observational study of children with diarrheal disease and a cohort study of healthy infants, both conducted in Ho Chi Minh City (HCMC), Vietnam [36, 37]. Both studies were approved by the institutional review boards of collaborating institutions HCMC and the Oxford Tropical Research Ethics Committee (OxTREC No. 1045-13) in the UK. Written informed consent by a legal guardian was a prerequisite for enrolment into the studies.

### Serum samples

Paired acute-convalescent serum samples were collected as a component of a prospective, observational, multicenter, cross-sectional study conducted in HCMC, Vietnam. The clinical and microbiological data from this study has been published previously [37]. For this investigation, we utilized acute serum samples collected from microbiologically confirmed *Shigella* (*n* = 33) or *Salmonella* (*n* = 24) cases when they first presented at hospital with acute diarrheal disease (patients bled prior to diagnostic testing) (Table 1). Convalescent (follow-up) serum samples collected at a follow-up visit, 4 weeks (± 1 week) after being enrolled in the study. Umbilical cord blood was collected from a large prospective birth cohort study, where healthy pregnant mothers who visited Hung Vuong Obstetrics Hospital in HCMC, Vietnam, were recruited prior to birth and cord blood sampled after delivery [36, 38]. Serum extracted from maternal blood during pregnancy has previously been subjected to ELISA to measure *S. sonnei* O-antigen IgG [39]; the cord blood samples screened here were from mothers with high (*n* = 45) and low (*n* = 40) *S. sonnei* O-antigen IgG titers (Table 1).

**Table 1:** Summary of genomes compared, serum samples tested, *Shigella* antigens studied, and in vivo immunogenicity testing conducted in present “reverse vaccinology 2.0” study

	Category		Number
***Genome comparison***	Number of genomes		**57**
		*S. sonnei*	2
		*S. flexneri*	4
		*S. dysenteriae*	2
		*S. boydii*	2
		Pathogenic *E. coli*	32
		Non-pathogenic *E. coli*	15
***No. of subjects***	*Shigella* patients		**33**
		*S. flexneri*	2
		*S. sonnei*	31
	*Salmonella* patients		**24**
	Newborn (cord serum)		**85**
		Low LPS titer	40
		High LPS titer	45
***Antigen microarray***	Number of expressed antigens		**234**
		*S. flexneri*-specific	22
		*S. sonnei*-specific	8
		*Shigella* orthologs	102 (× 2)
***Immunogenicity*** **in vivo**	Number of antigens tested		**8**

### Bioinformatic analysis

The complete chromosomal sequences of 10 *Shigella* and 47 *Escherichia coli* (*E. coli*) were retrieved from GenBank (accessed in July 2014 using an in-house script described in Supplementary file 1). The incorporated *Shigella* sequences included 2 *S. boydii*, 2 *S. dysenteriae*, 4 *S. flexneri*, and 2 *S. sonnei*. The collection of *E. coli* genomes used included both pathogenic (*n* = 32) and non-pathogenic variants (*n* = 15). A complete list of *E. coli* and *Shigella* genome sequences utilized in the present study is indicated in Additional File 2 Table S1. The CMG-Biotools (Comparative Microbial Genomics) workbench was used to identify the core genome of all sequences [40]. Protein sequences were extracted based on published annotations for all genomes. To identify conserved and unique regions of the genomes, we performed pairwise reciprocal BLASTP on all extracted protein sequences. Sequences were clustered into one orthologous group using the criteria of alignment of at least 50% similarity matches and alignment length of at least 50% of the longest sequence in the comparison.

By comparing the genomes, several protein subsets of interest were considered: (1) sequences present in all *S. sonnei* and absent from all *E. coli*, (2) sequences present in all *S. flexneri* and absent from all *E. coli*, and (3) sequences present in all *S. sonnei* and *S. flexneri* and absent from all *E. coli*. The output identified from each subset was manually curated by performing a BLASTN search of their sequences against the NCBI database. Since *Shigella* is phylogenetically nested within the *E. coli* species, they show a very low level of divergence in chromosomal genetic makeup. Hence, the number of proteins that fulfilled the above criteria (i.e., in groups 1 to 3) was limited (all are shown in Additional file 3 Table S2) and was not sufficient to develop downstream immunogenic assays.

We additionally included a subset of potentially immunogenic proteins present in both *S. sonnei* and *S. flexneri* genomes, notwithstanding their presence in the examined *E. coli* genomes. In brief, the annotation and protein sequences of each orthologous group were retrieved from the input *Shigella* genomes (using an in-house script described in Supplementary File 2). Sequences associated with mobile genetic elements (IS elements, transposases, and prophages, excluding pathogenicity islands) were manually checked and removed. Other proteins predicted to not be targets of antibody, by annotation of cellular function and location, were further excluded. These include proteins in toxin-antitoxin systems, bacterial conjugation, plasmid inheritance, genome replication, transcription or protein expression, cellular metabolism, and other cytoplasmic proteins of unspecified function. The retaining subset mostly consists of predicted outer membrane, secreted, periplasmic, and cell wall proteins (Additional file 5 Table S3). Unannotated (hypothetical) proteins were subjected to characterization in Pfam [41], the transmembrane domain prediction server TMHMM [42], and the signal peptide prediction server SignalP4.1 [43] (using default Gram-negative prokaryote settings). Proteins which may show potential immunogenicity (i.e., due to their location on the outer membrane, cell wall, possessing transmembrane domain(s), or possessing a signal peptide) were retained. Unannotated proteins identified by Pfam as bacteriophage-related were discarded.

### Protein microarray

Proteins selected through the bioinformatics pipeline are shown in Additional file 5 Table S3. These targets were expressed, printed, and probed as described previously for other protein microarray projects [44–46]. Briefly, the corresponding coding sequences from selected *Shigella* proteins were amplified, cloned into a pXT7 vector, and expressed using a high-throughput in vitro transcription/translation (IVTT) *E. coli* system (BiotechRabbit, GmbH). Controls lacking DNA were included to account for background reactivity with *E. coli*, where IVTT was conducted without plasmid DNA. Expressed *Shigella* antigens from IVTT reactions were printed onto nitrocellulose-coated glass GraceBio slides using an Omni Grid 100 microarray printer (Genomic Solutions). LPS from *Shigella* (Sigma) was also printed on the microarray slides to act as positive control. Slides (with *E. coli* lysate (McLab) at a final concentration of 1 mg/ml) were probed with human serum (diluted 1:200), followed by biotin-conjugated secondary antibodies specific for human IgM, IgG, and IgA (Jackson ImmunoResearch). Binding antibody was detected using streptavidin-conjugated SureLightH P-3 (Columbia Biosciences), measured using Perkin Elmer ScanArray Express HT microarray scanner. Spot intensities were quantified using the ScanArray software.

### Data analysis

Fold-over-control (FOC) normalizations were conducted to reduce assay to assay variation by dividing the mean spot intensities for each antigen by the intensity for the no DNA control IVTT. Positive serum reactivity to an antigen was defined as a FOC > 2 (i.e., > 2-fold increase in the mean intensity over the background control). Log2-transformed FOC values from paired acute and convalescent samples were compared using a Bayes regularized *t* test adapted from Cyber-T for protein arrays [47–49]. *p* values were subjected to Benjamini and Hochberg (BH) correction to control for false discovery rate [50]. Data were graphed using the R statistical software (http://www.r-project.org) and packages “Superheat,” “ggplot2,” “rgl,” and “fmsb.”

### Protein immunization

His-tagged variants of selected proteins (NmpC, FepA, HtrB, EmrK, NlpB, FhuA, CjrA, and MdtA) (Additional file 6 Table S4) were successfully expressed in a BacPowerTM *E. coli* protein expression system and purified using nickel affinity chromatography (GenScript Limited, Hong Kong). Four months old, male New Zealand rabbits (*n* = 2 per protein) were immunized with 0.2 mg of the successfully expressed and purified protein, and serum drawn at 1-week post-immunization of the third dose. The immunogenicity of each protein was assayed by testing the pre-immune and post-immune rabbit sera for sero-positivity using indirect enzyme-linked immunosorbent assay (ELISA) and immunoblot. For ELISA, plates were coated with protein at 4 μg/ml, blocked, incubated with sera (at 1 mg/ml IgG concentration), and detected using anti-rabbit IgG Fc-HRP secondary antibody. For western blots, 50 ng/well of purified proteins was run on SDS-PAGE, transferred to nitrocellulose membrane, blocked, probed with pre-immune and post-immune rabbit sera, and detected with goat anti-rabbit IgG-IRDye^800cw^ secondary antibody.

### Serum bactericidal assay

Purified serum antibody from immunized rabbits was tested for serum bactericidal activity (SBA) against *S. flexneri* 2a (strain EG 0478), *S*. *sonnei* (strain DE 1404, containing a cat chloramphenicol resistance gene on the virulence plasmid), and *S.* Typhimurium (strain ATCC 14028) using a previously described SBA protocol [51, 52]. Heat-inactivated sera were serially diluted from 50 to 0.07 μg/ml, then combined with bacteria (250 CFU/well) and 5 μl of baby rabbit complement and incubated at 37 °C for 90 min. Viable bacterial cells were estimated at time 0 min (T0) and at 90 min post-incubation (T90) by plating on nutrient agar plates. Bactericidal activity was calculated as a ratio of CFU at T90 over T0, from which SBA titers were estimated at 50% bactericidal activity. Convalescent immune serum from a confirmed *Shigella*-infected patient was used as a positive control [38]. All serum samples were tested in triplicate and the SBA titers averaged. The SBA assays with *S*. *sonnei* DE 1404 were performed with and without the supplementation of 10 μg/ml chloramphenicol. *S. sonnei* has the propensity to lose the virulence plasmid and O-antigen culture during culture, and the addition of 10 μg/ml chloramphenicol was to ensure the maintenance of plasmid and O-antigen during the SBA assay via the added cat gene. These data were compared to assess potential killing differences between plasmid+ and plasmid− organisms.

## Results

### Bioinformatic analysis identifies potential immunogenic *Shigella* core antigens

Genomic comparison of *Shigella* and *E. coli* core genomes was conducted with the aim of selecting both species-specific and species cross-reactive *Shigella* proteins common to the most globally dominant species, *S. flexneri* and *S. sonnei*. Protein sequences were extracted from the annotated chromosomes of various *Shigella* species (*n* = 10), pathogenic (*n* = 32) and non-pathogenic (*n* = 15) *E. coli* (Table 1 and Additional File 2 Table S1), and a list of potentially immunogenic antigens was selected using bioinformatic comparison, a list of potentially immunogenic antigens was selected (Table 2 and Additional File 5 Table S3). Our analysis was restricted to chromosomal proteins to identify novel immunogenic targets, as several proteins on *Shigella* virulence plasmids have already been extensively studied for their immunogenicity [53]. The analysis identified 22 *S. flexneri*-specific, 8 *S. sonnei*-specific, and 2 *Shigella*-specific proteins (IpaH3.1, IpaH4.5). Another 100 potentially immunogenic orthologs, from both *S. sonnei* and *S. flexneri*, were further included to expand the downstream immunogenic assays. *Shigella* LPS (O-antigen) was included as a positive control. This resulted in a total of 235 proteins that were expressed in vitro, and successfully printed on an antigen microarray for downstream analysis (Additional File 5 Table S3).

**Table 2:** Description of the protein subsets of interests studied during chromosomal genome comparison between *S. sonnei*, *S. flexneri*, and pathogenic and non-pathogenic *E. coli*

		Protein subset of interest	Excluded proteins	Primary result	Following first filtration^a^	Final selected proteins^b^
**Chromosome-encoded proteins**	**Proteins found in** ***S. sonnei***	1	Excluding proteins found in non-pathogenic *E. coli*	63	37	11
		2	Excluding proteins found in any other *Shigella* or *E. coli* spp.	25	16	8
	**Proteins found in** ***S. flexneri***	3	Excluding proteins found in non-pathogenic *E. coli*	48	29	20
		4	Excluding proteins found in any other *Shigella* or *E. coli* spp.	25	25	18
	**Proteins shared between** ***S. sonnei*** **and** ***S. flexneri***	5	Excluding proteins found in non-pathogenic *E. coli* chromosomes	8	1	1
	**Other predicted immunogenic** ***Shigella*** **proteins**	6				114

^a^First filtration: removal of insertion sequence (IS) elements, transposases, transposons, and bacteriophage-related proteins etc.

^b^Second filtration: removal of unspecific proteins (according to BLASTn), cytoplasmic and hypothetical proteins with bacteriophage domain

### Antigen microarray reveals broad seroconversion following *Shigella* infections

The *Shigella* antigen microarray allowed us to assess the IgM, IgA, and IgG responses against the selected antigens following symptomatic *Shigella* infections. The antigen microarray was probed for sero-reactivity with pairs of acute and early convalescent (i.e., 3- to 4-week follow-up) sera from microbiologically confirmed *Shigella*-infected diarrheal patients (*n* = 34) (Table 1). The present study used samples from patients infected with the two current globally dominant *Shigella* species, *S. flexneri* (*n* = 2) and *S. sonnei* (*n* = 32).

**Fig. 1:**
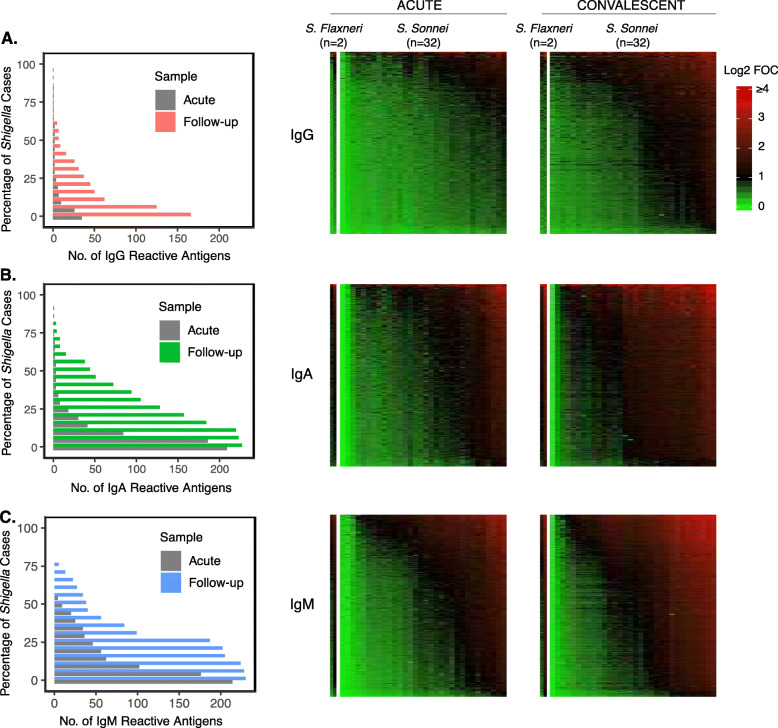
Sero-reactivity of *Shigella* antigens following *Shigella* infections. **a** IgG, **b** IgA, and **c** IgM reactivity was assessed in gastrointestinal disease patients during acute *Shigella* infection and at follow-up. IgG, IgA, and IgM responses (Log_2_ FOC) were graphically represented as heat maps, where *Shigella* antigens were ordered from bottom to top by increasing average responses and *Shigella*-infected patients were ordered from left to right by increasing average responses

*Shigella* infection led to widespread seroconversion in all measured antibody isotypes (IgG, IgA, and IgM) among individuals and across multiple tested antigens, as observed by the increase in sero-reactivity (i.e., measured as fold change over control greater than 2, FOC > 2) from acute to early convalescence (Fig. 1). IgG sero-reactivity (i.e., FOC > 2) analysis showed that at least one individual (3% of all patients) during acute and convalescent phase reacted to a maximum of 35 and 166 antigens, respectively (Fig. 1a). Additionally, 50% of the individuals (i.e., 17 patients) in acute and early convalescent phase sero-reacted (IgG) to 2 and 7 antigens, respectively (Fig. 1a).

The IgA responses were markedly higher than the IgG responses, with at least one individual generating a detectable IgA response to 209 and 227 antigens during acute disease and convalescence, respectively (Fig. 1b). We also observed that > 50% of cases in acute and convalescence produced a reactive IgA response to 3 and 44 antigens, respectively. IgM against the antigens also markedly increased between acute *Shigella* infections and convalescence (with 214 to 230 reactive antigens detected in at least 1 patient and 9 to 38 reactive antigens in > 50% of patients) (Fig. 1c).

We additionally observed that antibody responses were on average higher in patients with inflammatory (i.e., bloody diarrhea) as compared to non-inflammatory (i.e., watery diarrhea) disease (as can be seen in the scatter plots comparing mean IgG, IgA, and IgM responses at early convalescence, Additional File 7 Fig. S1). As a control for the assay, the antigen array was probed with paired acute and convalescent serum (*n* = 24) from diarrheal patients infected with an alternative genus of diarrheal pathogen, *Salmonella*. Notably, there were no significant increases in IgG, IgA, or IgM responses between the acute and convalescence in *Salmonella-*infected diarrheal cases, indicating that the antibody reactivity observed with the serum from the *Shigella*-infected patients was specific to *Shigella* (Additional File 8 Fig. S2).

### *Shigella* core antigen microarray identifies novel immunogenic antigens

**Fig. 2:**
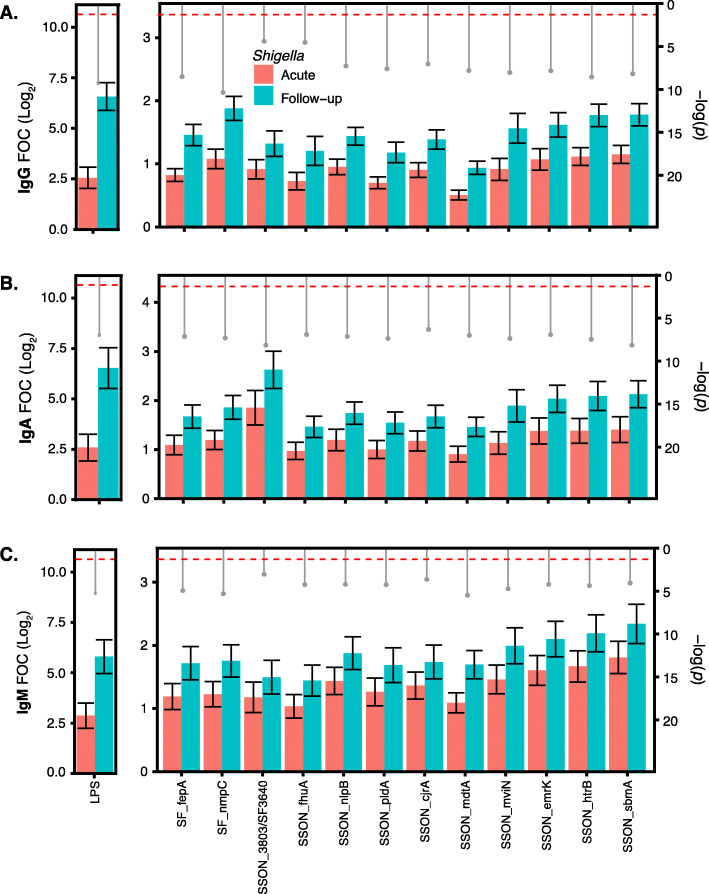
Sero-reactivity of twelve highly reactive *Shigella* antigens. Microarrays with *Shigella* antigens were probed for IgG, IgA, and IgM responses (Log2-transformed fold-over-control (FOC)) with acute and convalescent (or follow-up) sera from gastrointestinal patients with laboratory-confirmed *Shigella* infections. Mean IgG (**a**), IgA (**b**), and IgM (**c**) responses were compared between acute and follow-up samples from *Shigella*-infected patients, with *p* values (represented in gray drop-down pin heads) calculated using the Benjamini-Hochberg corrected Cyber-T test. Error bars (black) represent 95% confidence interval around the mean. The dashed horizontal line (red) indicates the position where *p* value = 0.05

**Fig. 3:**
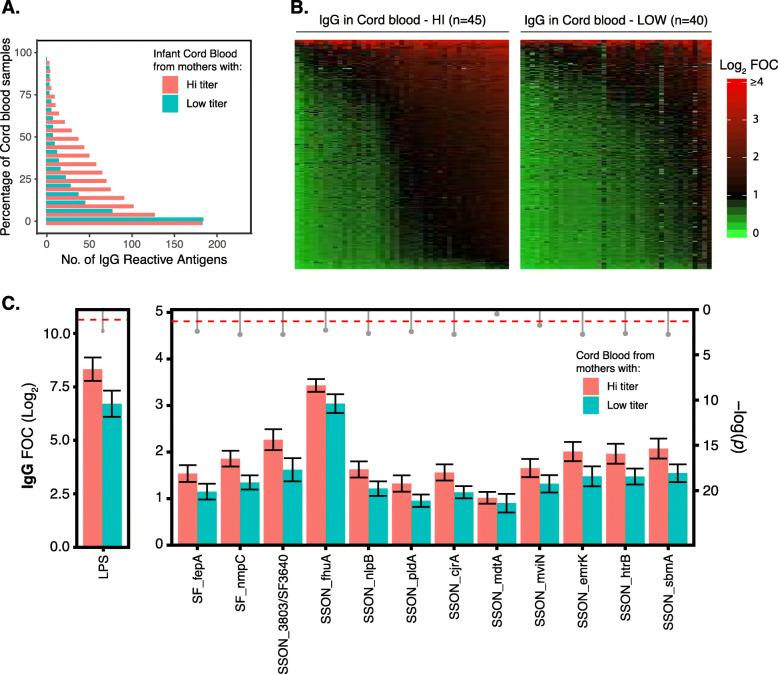
Reactivity of cord blood from mothers with high or low IgG titers to *Shigella* antigens. **a**, **b** IgG reactivity of cord blood to all tested *Shigella* antigens. In the heat maps, *Shigella* antigens were ordered from bottom to top by increasing average IgG reactivity and cord blood samples were ordered from left to right by increasing average IgG reactivity. **c** IgG reactivity to the top twelve highly reactive *Shigella* antigens was compared between cord blood from high and low IgG titer mothers using the Benjamini-Hochberg corrected Cyber-T test (*p* values represented in gray drop-down pin heads). Error bars (black) represent 95% confidence interval around the mean. The red dashed horizontal line is at *p* value = 0.05

As has been previously observed, the highest antibody responses at early convalescence following *Shigella* infections were against the *Shigella* LPS O-antigen. Additionally, we selected a subset of 12 immunogenic protein antigens using data generated by the antigen array microarray results; the criteria for this selection were the smallest *p* value (Benjamini-Hochberg corrected Cyber-T test) from comparison between acute and convalescent antibody responses, and the highest mean antibody responses (i.e., Log_2_(FOC) values) at early convalescence, etc. The twelve selected antigens were *NmpC* (SF_nmpC) and *FepA* (SF_fepA) from *S. flexneri*, and *HtrB* (SSON_htrB), *EmrK* (SSON_emrK), *NlpB* (SSON_nlpB), *FhuA* (SSON_fhuA), *CjrA* (SSON_cjrA), *MdtA* (SSON_mdtA), *SbmA* (SSON_sbmA), *MviN* (SSON_mviN), *PldA* (SSON_pldA), and 3803 (SSON_3803) from *S. sonnei*. Sero-reactivity between acute and early convalescence was compared using the Benjamini-Hochberg corrected Cyber-T test. For all 12 antigens, we observed a statistically significant (*p* < 0.05) increase in mean sero-reactivity between acute to early convalescence in all three antibody isotypes (Fig. 2). Furthermore, > 50% of the *Shigella*-infected cases at convalescence (or follow-up) had positive IgG, IgA, and IgM (i.e., FOC > 2) responses to all selected antigens, with the exception of SSON_mdtA and SSON_fhuA (Fig. 3).

*Shigella* poses the greatest health burden in children under the age of 2 years; therefore, an effective vaccine against *Shigella* needs to induce a protective immune response in young children. Hence, we assessed the antibody responses to the 12 selected antigens in children aged < 2 years (*n* = 8) in comparison to those aged > 2 years (*n =* 26). At early convalescence, there were no significant differences in both the mean antibody (IgG and IgA) sero-reactivity (Additional File 9 Fig. S3A and B) or the percentage of cases that showed positive antibody (IgG and IgA) responses (Fig. S3), when compared between age groups. These data demonstrate that younger children generate antibody responses to the 12 selected antigens. Additionally, we also compared sero-reactivity between male and females; all selected antigens elicited similar antibody responses (IgG and IgA) at early convalescence in both female (*n* = 20) and male (*n* = 14) patients with diarrhea (Fig. S3C and D).

### Transplacentally transferred antibodies

A potential mechanism for protecting young children and neonates is the prenatal vaccination of pregnant mothers [54]. Prenatal vaccines require the mother to mount a protective IgG response that can be efficiently transferred transplacentally to the unborn fetus [54]. Therefore, we measured the transplacental transfer of IgG as an indirect assessment of whether the selected immunogenic antigens could serve as potential prenatal vaccine candidates. We compared the IgG sero-reactivity at birth (cord blood serum) between infants from mothers with high (*n* = 45) and low (*n* = 40) antibody titers (to *Shigella* O-antigen) (Fig. 3). In general, infants from mothers with high antibody titers demonstrated greater IgG sero-reactivity against tested *Shigella* antigens in comparison to infants born to mothers with low IgG titers (Fig. 3). Specifically, with the exception of antigen SSON_mdtA, IgG sero-reactivity for the remaining 11 selected antigens was significantly greater (*p* < 0.05) in infants born to mothers with high antibody titers, than those with low antibody titers (Fig. 3c). These data show that 11/12 selected *Shigella* antigens are capable of generating IgG antibodies that can successfully transfer across the placenta in a concentration-dependent manner and may serve as good candidates for prenatal *Shigella* vaccines.

### Immunogenic testing of *Shigella* antigens

**Fig. 4:**
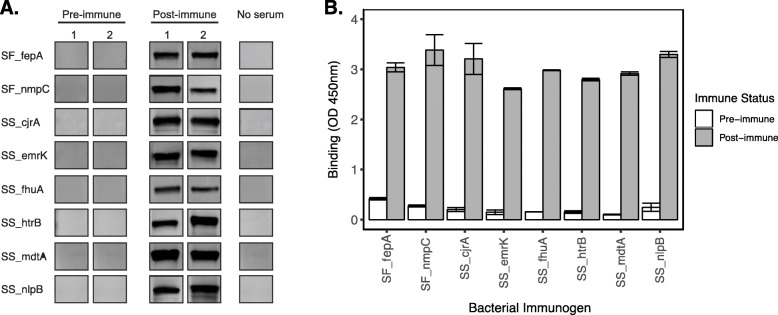
Immunogenicity testing of selected *Shigella* proteins in vivo. Rabbits were immunized with top reactive *Shigella* proteins, fepA, nmpC, cjrA, emrK, fhuA, htrB, mdtA, and nlpB. Positive antibody responses to respective *Shigella* antigen or immunogen in the immunized rabbits were tested using **a** western blot and **b** ELISA

We then proceeded to conduct preclinical testing of the top sero-reactive antigens. A BacPowerTM *E. coli* protein expression system was used to express SF_nmpC, SF_fepA, and SSON_htrB, SSON_emrK, SSON_nlpB, SSON_fhuA, SSON_cjrA, SSON_mdtA, SSON_sbmA, SSON_mviN, SSON_pldA, and SSON_3803. We were unsuccessful in expressing SSON_sbmA, SSON_mviN, SSON_3803, and SSON_pldA using this system (Additional File 6 Table S4). Therefore, we proceeded to test the remaining eight antigens (i.e., SF_nmpC, SF_fepA, and SSON_htrB, SSON_emrK, SSON_nlpB, SSON_fhuA, SSON_cjrA, and SSON_mdtA) in rabbits to ensure they could induce an antibody response when immunized as recombinant antigens. Rabbits were immunized separately with the eight antigens, and the serum was screened to measure binding IgG antibodies. All eight of the purified antigens induced robust IgG responses in vivo, which was detectable by immunoblot and ELISA (Fig. 4). We next tested the immunized rabbit sera for the potential to induce bacterial killing in an antibody-dependent complement-mediated serum bactericidal assay. We measured the killing potential of the purified rabbit serum against *S. flexneri*, *S. sonnei*, and *Salmonella* Typhimurium to ensure that the serum bactericidal activity (SBA) induced by the immunized antigens was *Shigella* specific. The SBA assay was unable to detect bactericidal activity below 50 μg/ml against *S. sonnei* (Table 1 and Additional File 10 Table S5) whether grown with or without chloramphenicol, which was used to maintain the virulence plasmid and O-antigen cluster in *S. Sonnei* due to the plasmid containing a *cat* resistance gene. However, six of the eight *Shigella* immunogens (i.e., SF_fepA, SSON_cjrA, SSON_emrK, SSON_fhuA, SSON_mdtA, and SSON_nlpB) induced antibody responses with strong serum bactericidal activity against *S. flexneri* (i.e., 50% SBA titer < 50 μg/ml) (Table 1); the lowest 50% SBA titer was induced by the immunogen SSON_cjrA (Table 3). Our results show that the antigen SSON_cjrA was the most potent inducer of bactericidal antibodies against *Shigella* and the best antigen to take forward into future testing as a potential vaccine antigen.

**Table 3:** Immunization with highly reactive *Shigella* proteins elicits protective antibody responses. Rabbits were immunized with 0.2 mg of *Shigella* proteins, and sera (at 1 week post-immunization) were tested in vitro for serum bactericidal activity (SBA) against *Shigella flexneri*, *Shigella sonnei*, and *Shigella* Typhimurium. SBA titers presented below are estimated at 50% bactericidal activity

	Post-immunization SBA titer (μg/ml)								SBA titer (dilution)
	fepA	nmpC	cjrA	emrK	fhuA	htrB	mdtA	nlpB	*Shigella*-immune sera
*Shigella flexneri*	0.4	> 50	0.0046	0.7	4.8	> 50	10.2	0.92	44,321
*Shigella sonnei*	> 50	> 50	> 50	> 50	> 50	> 50	> 50	> 50	29,561
*Salmonella* Typhimurium	> 50	> 50	> 50	> 50	> 50	> 50	> 50	> 50	> 50

## Discussion

In the current study, we exploited reverse vaccinology 2.0 to integrate both comparative genomics and human immuno-proteome analysis to identify novel immunogenic chromosomal *Shigella* proteins. Genomic comparison and bioinformatic analysis of 57 *Shigella* and *E. coli* genomes allowed us to narrow down to 235 predicted immunogenic antigens. The predicted antigens were then expressed and printed onto a microarray, probed with a panel of sera from *Shigella*-infected individuals, to narrow the selection to 12 highly sero-reactive antigens. We confirmed that antibody responses to these 12 antigens were similar across sex (i.e., males and females) and two age groups (i.e., < 2 years and > 2 years). Using cord blood samples, we additionally observed that IgG responses to 11 of these 12 antigens could be transmitted transplacentally, hence suggesting the possible application of these antigens as prenatal vaccine candidates. Among the 12 antigens, 8 were successfully expressed as recombinant proteins. Six of these antigens were both immunogenic in animal models and generated functionally protective antibody responses against *Shigella*. These were SF_fepA, SSON_cjrA, SSON_emrK, SSON_fhuA, SSON_mdtA, and SSON_nlpB, with SSON_cjrA being the most immunogenic in terms of eliciting antibody-mediated bactericidal responses. Such bactericidal effects against *S. sonnei* were not observed in vitro, probably due to the protection against complement-mediated killing afforded by its high molecular weight capsule [55]. However, the immunogenicity of these antigens suggests that during *S. sonnei* infections, the capsule can be modulated to expose these functional proteins in vivo. This highlights the complex pathogenesis of *S. sonnei* and the difficulty in developing a suitable vaccine candidate.

The biological functions of these six *Shigella* immunogenic proteins have been characterized previously. FepA and FhuA serve as outer membrane proteins which bind and transport siderophores (ferric enterobactin and ferrichrome, respectively) [56, 57]. CjrA shares substantial homology to *Pseudomonas aeruginosa* PhuW and potentially acts to sequester iron from heme, the most common iron source in mammals [58]. EmrK and MdtA are subunits of the multidrug efflux pump EmrKY and MdtABC, respectively [59, 60], which contribute to resistance to bile salt and antimicrobials in *E. coli*. In addition, EmrKY has been shown to confer *Shigella* survival in infected macrophages, facilitating its invasive pathogenesis in the human host [59]. NlpB forms part of the outer membrane protein (OMP) assembly complex, which assembles and inserts beta-barrel proteins into the outer membrane [61, 62]. The immunogenicity of these proteins points to their potentially high expression during *Shigella* infections, concurring with the survival strategies of pathogenic bacteria. Particularly, within-host iron is key to bacterial replication, and the ability to sequester and transport host iron is pivotal to the pathogenesis of *Klebsiella pneumoniae* [63] and *Staphylococcus aureus* [64].

None of the *Shigella* antigens (i.e., FepA, CjrA, EmrK, FhuA, MdtA, and NlpB) identified as immunogenic in the present study has been previously characterized for immunogenicity either in the context of natural *Shigella* infections or vaccination. Five of these antigens (i.e., FepA, CjrA, EmrK, FhuA, MdtA, and NlpB) are conserved in pathogenic *E. coli*, but they have not been tested for immunogenicity following pathogenic *E. coli* infection either. However, the presence of the genetic cluster *cjrABC-senB* has been previously linked to uropathogenic *E. coli* [65]. The immunogenicity of these six proteins may be predictable, since they are either surface exposed outer membrane or periplasmic proteins. Outer membrane particles of *Shigella* (Generalized Modules of Membrane Antigens—GMMA) have historically been thought to be highly virulent and immunomodulatory and are currently being developed as a vaccine immunogen [66]. GMMA-based Shigella vaccine, 1790GAHB, was shown to be immunogenic in human clinical trials [67]. Immunogenicity of GMMA vesicles has been attributed to the presence of LPS. However, proteomic analysis of the GMMA detected FepA and FhuA [68]. Based on our findings, it is plausible that other *Shigella* antigens, such as those identified in our current study, are may be partly responsible for the immunogenic properties of GMMA.

Development of a vaccine against *Shigella* faces many challenges, including the ability to protect against multiple *Shigella* species and to raise sustained mucosal immunity [10]. Fortunately, all the antigens, with the exception of CjrA, are conserved between *S. flexneri* and *S. sonnei*. Therefore, these antigens could be used to create a vaccine that protects against the two globally dominant *Shigella* species, which accounted for almost 90% of all *Shigella* cases in the Global Enteric Multicenter study (GEMS) [17]. Furthermore, in addition to strong IgG responses, all six antigens raised significant IgA responses, which is the dominant immunoglobulin at the mucosa. IgA-mediated protection has been explained by both preventing *Shigella* infection of host cells and downregulating inflammation and intestinal tissue pathology at infected sites [69, 70]. Although the current study measured IgA in serum, it has been previously shown that *Shigella*-specific serum IgA positively correlates with mucosal IgA in the stool [14]. Additionally, we observed that IgG specific to the six immunogenic *Shigella* antigens were capable of transplacental transfer, indicating that the antigens could additionally serve as prenatal vaccine candidates to protect neonates.

## Conclusions

*Shigella* infections cause over a quarter of a billion gastrointestinal infection cases globally per annum [2]. Despite the high public health burden, there is currently no licensed vaccine available to prevent *Shigella* diseases. At present, LPS is a key antigen for the development of a vaccine against *Shigella* [10, 71]. Here, we identified six novel immunogenic *Shigella* proteins that could serve as additional vaccine candidates or could be conjugated to O-antigens to provide some cross-protection. Future *Shigella* challenge studies in animal models or human controlled infection models are needed to test the potency of these identified six antigens as vaccine candidates alone or as new generation glycoconjugates.

## Supplementary Information

**Additional file 1: Supplementary file 1.** In-house script to retrieve complete *Shigella* and *Escherichia coli* chromosomal sequences from GenBank (accessed July 2014).

**Additional file 2: Table S1.** List of *Shigella* and *E. coli* strains used for the bioinformatics genome comparison.

**Additional file 3: Table S2.** Identified protein within subsets of interests during chromosomal genome comparison between *S. sonnei*, *S. flexneri*, pathogenic and non-pathogenic *E. coli. *

**Additional file 4: Supplementary file 2.** Script used to retrieve annotation and protein sequences from the input *Shigella* genomes.

**Additional file 5: Table S3.** List of Shigella proteins and antigens that were successfully tested on the Immunoassay protein microarray.

**Additional file 6: Table S4.** The recombinant expression of highly reactive *Shigella* proteins for in vivo immunogen testing.

**Additional file 7: Figure S1.** No statistically significant seroconversion to *Shigella* antigens detected in diarrheal patients during *Salmonella* infections.

**Additional file 8: Figure S2.** Antibody responses to natural *Shigella* infections are influenced by severity of disease.

**Additional file 9: Figure S3.** Convalescent antibody responses to top reactive *Shigella* antigens by age and sex.

**Additional file 10: Table S5.** Comparison of SBA titre from Rabbits immunized with *Shigella* candidate proteins using *Shigella* strain DE1404 grown with and without chloramphenicol (50mg/L).

## Data Availability

All data are presented in the manuscript (in the supplementary information), and raw data from gene selection from comparative genomics, signals from antigen array, and serum bactericidal data are freely available at 10.5281/zenodo.4320935 [72].

## Abbreviations

MSM: Men who have sex with men
LMICs: Low-middle income countries
MDR and XDR: Multi- and extensively drug resistant
WASH: Water, sanitation, and hygiene
CHIMs: Controlled human infection models
LPS: Lipopolysaccharide
HCMC: Ho Chi Minh City
IVTT: In vitro transcription/translation
FOC: Fold-over-control
BH: Benjamini and Hochberg correction
ELISA: Enzyme-linked immunosorbent assay
SBA: Serum bactericidal activity
OMP: Outer membrane protein
GMMA: Generalized Modules of Membrane Antigens
GEMS: Global Enteric Multicenter study
