# Supplementary material for: The identification of novel immunogenic antigens as potential Shigella vaccine components
Source: Genome Med. 2021 Jan 15;13:8. doi: 10.1186/s13073-020-00824-4 (PMC7809897; doi:10.1186/s13073-020-00824-4)
Supplement: Supplementary file 1 — Additional file 1: Supplementary file 1. In-house script to retrieve complete Shigella and Escherichia coli chromosomal sequences from GenBank (accessed July 2014). [file 13073_2020_824_MOESM1_ESM.docx]

**Supplementary File 1**. In-house script to retrieve complete *Shigella* and *Escherichia coli* chromosomal sequences from Genbank (accessed July 2014).

#!/usr/bin/env python

import sys, re, os, getopt

from Bio import Entrez

Entrez.email='haoct@oucru.org'

def Usage():

print('get_seq_by_acc.py')

print('Get sequence from NCBI using an accession number')

print('get_seq_by_acc.py [options]')

print('Options:')

print('-a <number>\t accession number')

print('-o <file name>\toutput fasta name')

print('-g <file name>\toutput genbank name')

print('-h\t\tshow this help')

def main():

try:

opts, args=getopt.getopt(argv, "ha:o:g:", ["help", "accession=", "output=", "genbank="])

except getopt.GetoptError as err:

print(str(err))

Usage()

sys.exit(2)

for opt, arg in opts:

if opt in ("-h"):

Usage()

sys.exit()

elif opt in ("-a"):

acc= arg

elif opt in ("-o"):

outfile = arg

elif opt in ("-g"):

outgb = arg

handle_fa=Entrez.efetch(db="nucleotide", id=acc, rettype="fasta", retmode="text")

handle_gb=Entrez.efetch(db="nucleotide", id=acc, rettype="gb", retmode="text")

#fastaname = str(outfile) + ".fasta"

#gbname = str(outfile) + ".gbk"

out_fasta=open(outfile, 'w')

out_fasta.write(handle_fa.read())

out_gb=open(outgb, 'w')

out_gb.write(handle_gb.read())

#out_handle.write(handle_gb.read())

out_fasta.close()

out_gb.close()

handle_fa.close()

handle_gb.close()

if __name__== "__main__":

argv=sys.argv[1:]

main()
