# Supplementary material for: The identification of novel immunogenic antigens as potential Shigella vaccine components
Source: Genome Med. 2021 Jan 15;13:8. doi: 10.1186/s13073-020-00824-4 (PMC7809897; doi:10.1186/s13073-020-00824-4)
Supplement: Supplementary file 2 — Additional file 2: Table S1. List of Shigella and E. coli strains used for the bioinformatics genome comparison. [file 13073_2020_824_MOESM2_ESM.docx]

**Table S1**. List of *Shigella* and *E.coli* strains used for the bioinformatics genome comparison.

|  | **Species*** | **Strain** |
| --- | --- | --- |
| *Shigella* | *S. boydii* | CDC 3083-94 |
|  |  | Sb227 |
|  | *S. dysenteriae* | 1617 |
|  |  | Sd197 |
|  | *S. flexneri Fxv* | 2002017 |
|  | *S. flexneri 2a* | 2457T |
|  |  | 301 |
|  | *S. flexneri 5* | 8401 |
|  | *S. sonnei* | 53G |
|  |  | Ss046 |
| Pathogenic *E.coli* | *E. coli* (AIEC) | LF82 |
|  |  | UM146 |
|  | *E. coli O83:H1* (AIEC, isolated from Crohn's) | NRG 857C |
|  | *E. coli* (Asymptomatic bacteriuria) | ABU 83972 |
|  | *E. coli* (Avian pathogenic E coli) | APEC 078 |
|  | E. *coli* (EAgEC) | 55989 |
|  |  | 42 |
|  | *E. coli* O26:H11 (EHEC) | 11368 |
|  | *E. coli* O127:H6 (EHEC) | E2348/69 |
|  | *E. coli* O157:H7 (EHEC) | EDL933 |
|  |  | sakai |
|  |  | Xuzhou21 |
|  | *E. coli* (EIEC) | 53638 |
|  | *E. coli* O55:H7 (EPEC) | CB9615 |
|  |  | RM12579 |
|  | *E. coli* (ETEC) | E24377A |
|  |  | H10407 |
|  | *E. coli* (Ethanologenic) | LY180 |
|  | *E. coli* (ExPEC) | PMV-1 |
|  |  | IHE3034 |
|  |  | S88 |
|  | *E. coli* (porcine ETEC) | UMNK88 |
|  | *E. coli* O104:H4 (Shiga toxin producing EAgEC) | 2009EL-2050 |
|  |  | 2011C-3493 |
|  | *E. coli* (STEC) | O103:H2 |
|  | *E. coli* (UPEC) | CFT073 |
|  |  | JJ1886 |
|  |  | NA114 |
|  |  | O7:K1 CE10 |
|  |  | clone D i14 |
|  |  | UMN026 |
|  |  | UTI89 |
| Non-Pathogenic *E.coli* | *E. coli* (B-strain lab) | BL21 DE3 |
|  |  | B/REL606 |
|  | *E. coli* (commensal) | BL21-Gold(DE3) pLysS AG |
|  |  | HS |
|  |  | IAI1 |
|  |  | SE11 |
|  |  | SE15 |
|  |  | ED1a |
|  | *E. coli* (environmental, free living) | SMS-3-5 |
|  | *E. coli* (K-12 lab strain) | K-12/ DH1 |
|  |  | K-12 / DH10B |
|  |  | K-12 / MG1655 |
|  |  | K-12 / W3110 |
|  | *E. coli* (Lab strain) | ATCC 8739 |
|  |  | P12b |

* AIEC = adherent-invasive E coli, EAgEC = enteroaggregative E. coli, EHEC = Enterohemorrhagic Escherichia coli, EIEC = Enteroinvasive Escherichia coli, EPEC = Enteropathogenic Escherichia coli, ETEC = Enterotoxigenic Escherichia coli, ExPEC = Extra-intestinal pathogenic Escherichia coli, STEC = Shiga toxin-producing Escherichia coli, UPEC = uropathogenic E. coli.
