# Supplementary material for: The identification of novel immunogenic antigens as potential Shigella vaccine components
Source: Genome Med. 2021 Jan 15;13:8. doi: 10.1186/s13073-020-00824-4 (PMC7809897; doi:10.1186/s13073-020-00824-4)
Supplement: Supplementary file 3 — Additional file 3: Table S2. Identified protein within subsets of interests during chromosomal genome comparison between S. sonnei, S. flexneri, pathogenic and non-pathogenic E. coli. [file 13073_2020_824_MOESM3_ESM.docx]

**Table S2.** Identified protein within subsets of interests during chromosomal genome comparison between *S. sonnei*, *S. flexneri*, pathogenic and non-pathogenic *E. coli.*

| **Proteins common to all studied S. sonnei chromosomes, absent from all studied Shigella and non-pathogenic E. coli chromosomes** | | |
| --- | --- | --- |
| **Locus tag** | **Gene name** | **Product** |
| SSON_0406 | Hypothetical | Hypothetical protein |
| SSON_1917 | Hypothetical | Hypothetical protein |
| SSON_2657 | Hypothetical | Hypothetical protein |
| SSON_2662 | cjrA | Iron-regulated protein |
| SSON_2663 | cjrB | Colicin Js-sensitive receptor protein |
| SSON_2664 | cjrC | Colicin Js-sensitive receptor protein |
| SSON_2665 | SenB | Enterotoxin |
| SSON_4110 | Hypothetical | Hypothetical protein |
| **Proteins common to all studied S. flexneri chromosomes, absent from all studied Shigella and non pathogenic E. coli chromosomes.** | | |
| **Locus tag** | **Gene name** | **Product** |
| SF0205 | Hypothetical | Type VI secretion system lysozyme-like sciD protein |
| SF0266 | Hypothetical | Hypothetical protein, putative 1TM and PAAR motif domain |
| SF0267 | Hypothetical | Hypothetical protein |
| SF0268 | Hypothetical | Hypothetical protein |
| SF0364 | yabO | Hypothetical protein, putative signal peptide (cleavage site between pos. 22&23) |
| SF0365/SF3265 | Hypothetical | Hypothetical protein, putative signal peptide (cleavage site between pos. 19&20) |
| SF0594 | Hypothetical/rshE | Hypothetical protein, putative 2TM domain |
| SF2093 | Hypothetical | Hypothetical protein, putative 5TM domain |
| SF2097 | rfc | O-antigen polymerase |
| SF2098 | rfbG | dTDP-rhamnosyl transferase |
| SF2099 | rfbF | dTDP-rhamnosyl transferase |
| SF2100 | rfbE | Polysaccharide biosynthesis protein |
| SF2499 | Hypothetical | Amino acid transporter |
| SF3508 | Hypothetical | Hypothetical protein, putative 1TM and transcriptional regulatory protein domain |
| SF3510 | Hypothetical | Hypothetical protein/multidrug resistance protein MdtG, putative signal peptide (cleavage site between pos. 19&20) |
| SF3700 | shiB | Hypothetical protein |
| SF4362 | Hypothetical | Hypothetical protein |
| SF4376 | Hypothetical | Hypothetical protein, putative beta-lactamase superfamily domain |
| **Proteins common to all studied S. sonnei and S. flexneri chromosomes and absent from non-pathogenic E. coli** | | |
| SSON_1317/SF1383 | ipaH3 | Invasion plasmid antigen |
