# Supplementary material for: The identification of novel immunogenic antigens as potential Shigella vaccine components
Source: Genome Med. 2021 Jan 15;13:8. doi: 10.1186/s13073-020-00824-4 (PMC7809897; doi:10.1186/s13073-020-00824-4)
Supplement: Supplementary file 4 — Additional file 4: Supplementary file 2. Script used to retrieve annotation and protein sequences from the input Shigella genomes. [file 13073_2020_824_MOESM4_ESM.docx]

**Supplementary File 2**. Script used to retrieve annotation and protein sequences from the input *Shigella* genomes.

#!/usr/bin/perl

use strict;

use Bio::SeqIO;

my $fileGB = $ARGV[0];

my %hashgi;

my $nsec = 0;

my $seqio_object = Bio::SeqIO->new(-file => $fileGB);

my $nfeat = 0;

open (ROB, ">$fileGB.fna") or die ("Cannot open outputfile: $fileGB.fna\n");

open (SOL, ">$fileGB.fsa") or die ("Cannot open outputfile: $fileGB.fsa\n");

while (my $seq_object = $seqio_object->next_seq){

my $NC = $seq_object->accession_number;

for my $feat_object ($seq_object->get_SeqFeatures) {

$nfeat++;

my $sequence_string = $feat_object->spliced_seq->seq;

my $GI = 'not found';

my $PID = 'not found';

my $gene = 'not found';

my $locus_tag = 'not found';

my $translation = 'not found';

if ($feat_object->primary_tag eq "CDS"){

for my $tag ($feat_object->get_all_tags) {

if ($tag eq 'db_xref'){

for my $value ($feat_object->get_tag_values($tag)) {

if ($value =~ /GI:(\d+)/){

$GI = $1;

}

}

} elsif ($tag eq 'translation'){

for my $value ($feat_object->get_tag_values($tag)) {

if ($value =~ /(.+)/){

$translation = $1;

}

}

} elsif ($tag eq 'locus_tag'){

for my $value ($feat_object->get_tag_values($tag)) {

if ($value =~ /(.+)/){

$locus_tag = $1;

}

}

}

}

if ($locus_tag ne 'not found'){

print ROB (">$locus_tag.$fileGB\n$sequence_string\n");

print SOL (">$locus_tag.$fileGB\n$translation\n");

$nsec++;

}

}

}

}

close (SOL);

close (ROB);
