# Supplementary material for: The identification of novel immunogenic antigens as potential Shigella vaccine components
Source: Genome Med. 2021 Jan 15;13:8. doi: 10.1186/s13073-020-00824-4 (PMC7809897; doi:10.1186/s13073-020-00824-4)
Supplement: Supplementary file 6 — Additional file 6: Table S4. The recombinant expression of highly reactive Shigella proteins for in vivo immunogen testing. [file 13073_2020_824_MOESM6_ESM.docx]

**Table S4.** The recombinant expression of highly reactive *Shigella* proteins for *in vivo* immunogen testing.

| **Protein** | **Signal peptide**  **(aa)** | **Total length** | **Region Expressed** | **Protein Expression*** | **Note** |
| --- | --- | --- | --- | --- | --- |
| SF_nmpC | 1-23 | 360 | 24-360 | Successful |  |
| SF_fepA | 1-22 | 746 | 23-746 | Successful |  |
| SS_htrB | No | 306 | 54-306 | Successful |  |
| SS_emrK | N (1-33) | 387 | 36-387 | Successful |  |
| SS_nlpB | No | 345 | 29-345 | Successful |  |
| SS_fhuA | 1-33 | 729 | 34-729 | Successful |  |
| SS_cjrA | No | 305 | 33-230 | Successful |  |
| SS_mdtA | No | 413 | 25-413 | Successful |  |
| SS_sbmA | No | 406 | 161-206, 262-308, 360-406 | Unsuccessful |  |
| SS_mviN | No | 511 | 46-81 | Unsuccessful |  |
| SS_3803 | 1-52 | 1615 | 53-1615 | Unsuccessful | Only a small portion of this protein can be expressed |
| SS_pldA | 1-20 | 289 | 21-289 | Unsuccessful | Many transmembrane helices, so difficulty expressing |

*All recombinant proteins were expressed in an E.coli expression system by Genescript (Hong Kong) Limited according to provided protein sequences.
