# Supplementary material for: The identification of novel immunogenic antigens as potential Shigella vaccine components
Source: Genome Med. 2021 Jan 15;13:8. doi: 10.1186/s13073-020-00824-4 (PMC7809897; doi:10.1186/s13073-020-00824-4)
Supplement: Supplementary file 7 — Additional file 7: Figure S1. No statistically significant seroconversion to Shigella antigens detected in diarrheal patients during Salmonella infections. [file 13073_2020_824_MOESM7_ESM.docx]

**Figure S1**. No statistically significant seroconversion to *Shigella* antigens detected in diarrheal patients during *Salmonella* infections. *Shigella* antigens were probed for IgG, IgA and IgM responses with acute and convalescent (or follow-up) sera from diarrheal patients with laboratory confirmed-*Salmonella* infections. **A**) IgG, IgA and IgM reactivity were graphed as heatmaps, where *Shigella* antigens were ordered from bottom to top by increasing average responses and *Salmonella*-infected patients were ordered from left to right by increasing average responses. Mean IgG (**B**), IgA (**C**) and IgM (**D**) responses were compared between acute and follow-up samples from *Salmonella*-infected patients, with p-values calculated using the Benjamini Hochberg corrected cyber T test. Error bars (black) represent 95% confidence interval around the mean. The red dashed horizontal line is at *p*-value = 0.05.
