# Supplementary material for: The identification of novel immunogenic antigens as potential Shigella vaccine components
Source: Genome Med. 2021 Jan 15;13:8. doi: 10.1186/s13073-020-00824-4 (PMC7809897; doi:10.1186/s13073-020-00824-4)
Supplement: Supplementary file 8 — Additional file 8: Figure S2. Antibody responses to natural Shigella infections are influenced by severity of disease. [file 13073_2020_824_MOESM8_ESM.docx]

**
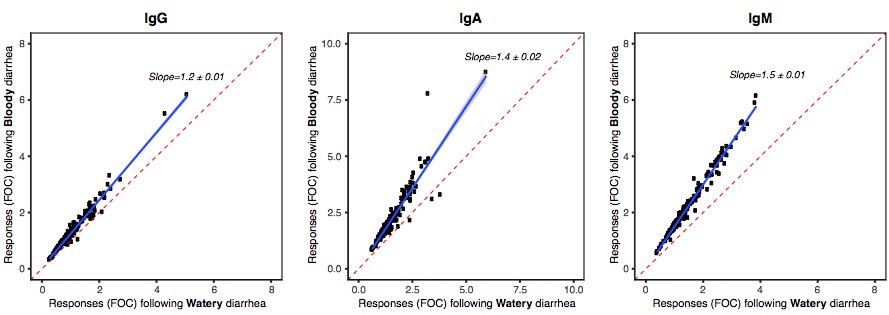
**

**Figure S2**. Antibody responses to natural *Shigella* infections are influenced by severity of disease. Convalescent IgG, IgA and IgM responses (FOC) to *Shigella* antigens were higher in patients who experienced severe diarrhea (i.e. bloody diarrhea as opposed to watery diarrhea). Red dashed diagonal line indicates the *y=x* position. The slope indicates the linear regression coefficient (with standard error) between mean antibody responses to each *Shigella* antigen in bloody versus watery diarrhea patients.
