# Supplementary material for: The identification of novel immunogenic antigens as potential Shigella vaccine components
Source: Genome Med. 2021 Jan 15;13:8. doi: 10.1186/s13073-020-00824-4 (PMC7809897; doi:10.1186/s13073-020-00824-4)
Supplement: Supplementary file 9 — Additional file 9: Figure S3. Convalescent antibody responses to top reactive Shigella antigens by age and sex. [file 13073_2020_824_MOESM9_ESM.docx]

**Figure S3.** Convalescent antibody responses to top reactive *Shigella* antigens by age and sex.

Mean IgG and IgA responses (Log2-transformed fold over control (FOC)) of follow-up serum from confirmed-*Shigella* infected patients to highly reactive *Shigella* antigens were analysed by age (A-B) and sex (C-D). Patients were grouped either by sex (i.e. female, *n*=20 and male, *n*=14) or by age (<2 yr, *n*=8 and ≥2yr, *n*=26). Error bars (black) represent 95% confidence interval around the mean.
