# Supplementary material for: The identification of novel immunogenic antigens as potential Shigella vaccine components
Source: Genome Med. 2021 Jan 15;13:8. doi: 10.1186/s13073-020-00824-4 (PMC7809897; doi:10.1186/s13073-020-00824-4)
Supplement: Supplementary file 10 — Additional file 10: Table S5. Comparison of SBA titre from Rabbits immunized with Shigella candidate proteins using Shigella strain DE1404 grown with and without chloramphenicol (50mg/L). [file 13073_2020_824_MOESM10_ESM.docx]

**Table S5.** Comparison of SBA titre from Rabbits immunized with *Shigella* candidate proteins using *Shigella* strain DE1404 grown with and without chloramphenicol (50mg/L).

|  | Post-immunization SBA titre (μg/ml) | | | | | | | | SBA titre (dilution) |
| --- | --- | --- | --- | --- | --- | --- | --- | --- | --- |
|  | fepA | nmpC | cjrA | emrK | fhuA | htrB | mdtA | nlpB | *Shigella*-immune sera |
| *Without*  *Chloramphenicol* | >50 | >50 | >50 | >50 | >50 | >50 | >50 | >50 | 29561 |
| *with Chloramphenicol* | >50 | >50 | >50 | >50 | >50 | >50 | >50 | >50 | 8580 |
